# Housing status is protective of neuropsychiatric symptoms among dementia-free multi-ethnic Asian elderly

**DOI:** 10.1186/s12877-024-05203-x

**Published:** 2024-08-23

**Authors:** Haoran Zhang, Yuwei Wang, Yaping Zhang, Saima Hilal, Ching-Yu Cheng, Tien Yin Wong, Christopher Chen, Narayanaswamy Venketasubramanian, Xin Xu

**Affiliations:** 1 School of Public Health and The 2nd Affiliated Hospital of School of Medicine, Zhejiang University, Hangzhou, Zhejiang 310000 China; 2 The Key Laboratory of Intelligent Preventive Medicine of Zhejiang Province, Hangzhou, Zhejiang China; 3 Ng Teng Fong General Hospital, Singapore, Singapore; 4 Memory, Ageing and Cognition Centre, Department of Pharmacology, Yong Loo Lin School of Medicine, National University of Singapore, Singapore, Singapore; 5 Centre for Innovation and Prevision Eye Health, Yong Loo Lin School of Medicine, National University of Singapore, Singapore, Singapore; 6 Department of Ophthalmology, Yong Loo Lin School of Medicine, National University of Singapore, Singapore, Singapore; 7 Singapore Eye Research Institute, Singapore National Eye Centre, Singapore, Singapore; 8 Tsinghua Medicine, Tsinghua University, Beijing, China; 9 Raffles Neuroscience Centre, Raffles Hospital, Singapore, Singapore

**Keywords:** Neuropsychiatric symptoms, Cognitive impairment, Socioeconomic status, Housing

## Abstract

**Background:**

Housing has been associated with dementia risk and disability, but associations of housing with differential patterns of neuropsychiatric symptoms (NPS) among dementia-free older adults remain to be explored. The present study sought to explore the contribution of housing status on NPS and subsyndromes associated with cognitive dysfunction in community-dwelling dementia-free elderly in Singapore.

**Methods:**

A total of 839 dementia-free elderly from the Epidemiology of Dementia in Singapore (EDIS) study aged ≥ 60 were enrolled in the current study. All participants underwent clinical, cognitive, and neuropsychiatric inventory (NPI) assessments. The housing status was divided into three categories according to housing type. Cognitive function was measured by a comprehensive neuropsychological battery. The NPS were assessed using 12-term NPI and were grouped into four clinical subsyndromes: psychosis, hyperactivity, affective, and apathy. Associations of housing with composite and domain-specific Z-scores, as well as NPI scores, were assessed using generalized linear models (GLM). Binary logistic regression models analysed the association of housing with the presence of NPS and significant NPS (NPI total scores ≥ 4).

**Results:**

Better housing status (5-room executive apartments, condominium, or private housing) was associated with better NPS (OR = 0.49, 95%CI = 0.24 to 0.98, *P* < 0.05) and significant NPS profile (OR = 0.20, 95%CI = 0.08 to 0.46, *P* < 0.01), after controlling for demographics, risk factors, and cognitive performance. Compared with those living in 1–2 room apartments, older adults in better housing had lower total NPI scores (*β*=-0.50, 95%CI=-0.95 to -0.04, *P* = 0.032) and lower psychosis scores (*β*=-0.36, 95%CI=-0.66 to -0.05, *P* = 0.025), after controlling for socioeconomic status (SES) indexes. Subgroup analysis indicated a significant correlation between housing type and NPS in females, those of Malay ethnicity, the more educated, those with lower income, and those diagnosed with cognitive impairment, no dementia (CIND).

**Conclusions:**

Our study showed a protective effect of better housing arrangements on NPS, especially psychosis in a multi-ethnic Asian geriatric population without dementia. The protective effect of housing on NPS was independent of SES and might have other pathogenic mechanisms. Improving housing could be an effective way to prevent neuropsychiatric disturbance among the elderly.

**Supplementary Information:**

The online version contains supplementary material available at 10.1186/s12877-024-05203-x.

## Introduction

Neuropsychiatric symptoms (NPS) are a heterogeneous group of noncognitive symptoms and behaviours involved in neurodegenerative dementias [1] and include psychosis, hyperactivity, apathy, and affective subsyndromes [2]. NPS are prevalent among the community-dwelling geriatric population [3], conferring a greater risk of cognitive decline and development of dementia [4]. A growing body of evidence has suggested that NPS could be early manifestations of prodromal or emergent dementia [5]. Presence of NPS [6], even of mild severity [7], carries a risk of progression from mild cognitive impairment (MCI) to all-cause dementia [8].

Housing, as a critical social determinant of health, plays a key role in dementia [9, 10] and disability [11]. A recent study demonstrated that neighbourhood disadvantage was associated with a higher risk of dementia among US older veterans [12]. Housing disadvantage, such as overcrowding, physical housing conditions, and household instability, affects not only cognitive decline, but also mental health [13, 14]. On the one hand, housing is positively correlated with socioeconomic status (SES) and is often used as a proxy for income [15]. Compared to younger people, older adults are more likely to stay at home and their health is more influenced by home environmental factors such as adequate sunshine, ventilation, and the size of living areas [16–18]. Past studies focused on younger population might have underestimated the direct or non-SES role of housing on mental health. On the other hand, although previous studies have shown that housing environment was associated with depressive and anxiety symptoms, as well as sense of happiness among general older adults [17, 19, 20], it remains unknown if housing status would be associated with differential patterns of NPS, especially among older adults at varying cognitive status before the occurrence of dementia. Studies have been conducted to identify factors that might affect NPS, including age, sex, ethnicity, and SES. For instance, increasing age, female sex, Malay ethnicity, a lower level of education, occupation, and income may be possible risk factors for the development of psychosis among older adults without dementia [21–24]. Differences in cognitive status are also important to investigate because the prevalence of NPS was higher even in the prodromal stages of dementia [25, 26]. However, it remains unclear whether the association between housing status and NPS differs by diverse demographic and cognitive status.

The extensive intervention of the Singapore government in regulating housing supply and demand provides a clear division of housing, from heavily subsidized public housing provided by the Housing and Development Board (HDB) to private housing [27]. Singapore was made up of three main ethnic groups: Chinese (74.0%), Malays (13.5%), and Indians (9.0%) [28]. Multiple ethnicities can be studied in a relatively uniform environment with similar exposures [29].

Hence, in the present study, we aimed to evaluate the effect of housing status on NPS and subsyndromes among dementia-free elderly adults in a community-dwelling Singaporean population. We hypothesized that (1) higher level of housing status is protective of NPS; (2) the effect of housing on NPS differs by demographic, SES, and cognitive status; (3) housing status would be associated with specific patterns of NPS subsyndromes.

## Methodology

### Study population

The Epidemiology of Dementia in Singapore (EDIS) study recruited participants from the Singapore Epidemiology of Eye Disease (SEED) study, a study comprised the Singapore Chinese Eye Study from 2009 to 2011, the Singapore Malays Eye Study from 2010 to 2013, and the Singapore Indian Eye Study from 2013 to 2015 [30]. The study was conducted face-to-face at the Singapore Eye Research Institute in Singapore. Participants in SEED who were 60 years and older were screened using the Abbreviated Mental Test (AMT) and self-reported history of forgetfulness. Positive screening was defined using these criteria: an AMT score of ≤ 6 for participants with six or less years of formal education; or an AMT score of ≤ 8 for participants with more than six years of formal education; or if the caregiver reported progressive forgetfulness. 1598 participants who screened positive were invited for the second phase of the study, of which 957 participants consented to participate. Written informed consent was obtained in the preferred language of participants and caregivers by bilingual study coordinators before the recruitment into the study, and ethics approval was obtained from both the Singapore Eye Research Institute and the National Healthcare Group Domain-Specific Review Board [31]. Ineligible participants were excluded during recruitment and exclusion criteria were major psychiatric illness or substance abuse disorder according to Diagnostic and Statistical Manual of Mental Disorders, Fourth Edition (DSM-IV), criteria; a malignant disease such as cancer, tumour, etc.; significant visual and auditory abnormalities, diagnosis of dementia (DSM-IV) [30].

### Vascular profile

Cardiovascular risk factors and demographic data, including age, sex, ethnicity, alcohol and smoking status, past medical history of diabetes mellitus, hypertension, hyperlipidaemia, and cardiovascular disease were collected. Smoking status was categorized into non-smokers and smokers (past and current smokers), and alcohol status was categorized into non-drinkers and drinkers (past and current drinkers). Hypertension diagnosis was defined as systolic blood pressure ≥ 140 mmHg and/or diastolic blood pressure ≥ 90 mmHg measured by a digital automatic blood pressure machine (OMRON-HEM 7203, Japan), or the use of anti-hypertensive medications. Diabetes mellitus diagnosis was defined as glycated haemoglobin ≥ 6.5% or using diabetic medications. A diagnosis of hyperlipidaemia was defined as total cholesterol levels ≥ 4.14 mmol/l, or the use of lipid-lowering medication. Cardiovascular disease was defined as documented history of myocardial infarction, congestive heart failure, atrial fibrillation, coronary angioplasty, or stenting.

### Housing status

Housing status was grouped into three categories: (1) 1–2 room HDB apartments; (2) 3–4 room HDB apartments; (3) privileged housing: 5-room executive HDB apartments, condominium, or private housing.

### Socioeconomic status assessment

A detailed questionnaire was administered to older adult participants on three common socioeconomic indicators [32–34] including education, current occupation, current income from all sources such as wages, pensions, and any other income, which were grouped into three categories respectively, according to established criteria and study data: Education- (1) no formal education; (2) primary education; (3) secondary and above education. Occupation was categorized as skilled (e.g., professional, legislator, and senior official), semiskilled (e.g., service worker, production craftsman, plant and machine operator, transportation driver, security guard), unskilled (e.g., agricultural worker, housekeeping and cleaning worker, labourer, odd job worker), and retired. For unemployed participants, the present study didn’t classify their occupation information based on their last occupation held. Instead, unemployed participants were included under the unskilled and unemployed group, according to the classification from previous literature [35, 36]. Income- (1) less than 1000 Singaporean dollar (SGD) a month; (2) more than or equal to 1000 SGD and less than 2000 SGD a month; (3) more than or equal to 2000 SGD a month.

### Quality control

In the study, not all participants speak the English language. The questionnaire was administered in English or translated into Malay/Tamil/Mandarin/Chinese dialects, and back-translated into English (based on the participant’s choice) by two different fluently bilingual interpreters. During administration, the participant was given a choice to be interviewed in either Malay/Tamil/Mandarin/Chinese dialects or English. All our interviewers were fluently bilingual. The study ensured that translations, trainings, or administration were done in a controlled and consistent manner through the following approaches: First, all translators and raters are multilingual speakers, and had relevant experience with medical research. Second, translation and training for investigations were conducted by a group of experts, experienced researchers, bilingual/multi-lingual clinical coordinators, and medical doctors, to ensure the quality of translation and back-translation of questionnaires, as well as multi-lingual data collection process. All study raters were bilingual/multilingual. Then, rating training was done through multiple steps which lasts for one month before a new researcher can start data collection. Only researchers who pass all training procedures can be independent raters for the study. This is the standardized data collection training procedure for all past and ongoing studies under the research centre in multi-ethnic and multi-lingual Singaporean populations [30, 31]. With the participant’s consent, randomly selected 5% of the interviews were recorded for periodic review by the investigators in the first month of each ethnic-specific sub-study for quality control (the Singapore Chinese Eye Study from 2009 to 2011, the Singapore Malays Eye Study from 2010 to 2013, and the Singapore Indian Eye Study from 2013 to 2015). Review criteria for raters include verbal fluency, accuracy of wording and adherence to administration instructions [37, 38]. The long-term program was inspected regularly by the Institutional Review Board (IRB) and the National Medical Research Council (NMRC) as per requirement.

### Cognitive assessment

All participants underwent the Montreal Cognitive Assessment (MoCA), as well as an extensive neuropsychological battery previously validated in Singapore [31]. The formal neuropsychological battery consisted of a number of subtests in five non-memory and two memory-specific domains, as follows [30]:

- Executive function [Frontal Assessment Battery and Maze Task].
- Attention [Digit Span, Visual Memory Span and Auditory Detection test].
- Language [Boston Naming Test and Verbal Fluency Test].
- Verbal memory [Word List Recall and Story Recall].
- Visual memory [Picture Recall and Weschler Memory Scale-Revised (WMS-R) Visual Reproduction Tests]
- Visuomotor speed [Symbol Digit Modality Test and Digit Cancellation Test].
- Visuoconstruction [WMS-R Visual Reproduction Copy task and Clock Drawing and the Weschler Adult Intelligence Scale-Revised (WAIS-R) subtest of Block Design].

For each participant, raw scores from each subtest were transformed into standardized Z-scores using the mean and standard deviation (SD) of that subtest. Subsequently, domain-specific Z-scores were calculated by averaging the Z-scores of the individual tests within that domain, and standardized using the mean and SD within that domain. Finally, a composite Z-score was computed by averaging the seven domain-specific mean Z-scores, which were also standardized using the corresponding mean and SD. The composite Z-score reflected global cognitive functioning, and higher scores indicate better cognitive performance [31]. Participants failed a test if their scores were below the education-adjusted threshold of 1.5 SD. Impairment in a domain was defined as failure in at least half of the tests in that domain. Weekly consensus meetings were held to make cognitive status of participants. “No cognitive impairment (NCI)” was defined as normal cognitive functioning on the comprehensive neuropsychological test battery. “Cognitive impairment, no dementia (CIND)” was defined as the absence of significant independence in daily activities and at least one impairment domain of the formal neuropsychological battery. CIND was further classified into mild (≤ 2 impaired domains) and moderate (> 2 impaired domains).

### Neuropsychiatric assessment

The 12-item neuropsychiatric inventory (NPI) was administered to reliable informants with frequent interactions with the study participant for at least 10 h a week, such as spouse, child, and close relative [39]. Briefly, the NPI comprised 12 psychological and behavioural symptoms and investigated the frequency and severity of these symptoms. A total score for each of these 12 symptoms would be derived by multiplying the severity and frequency. Individual symptoms were grouped into four clinical subsyndromes, and the grouping method has been validated in previous Singapore study population [40], as follows:

- Psychosis: Hallucinations, delusions, and nighttime behaviours;
- Hyperactivity: Agitation, disinhibition, elation, irritability, and aberrant motor behaviours;
- Affective: Depression and anxiety;
- Apathy: Apathy and appetite/eating behaviours.

The total score for each subsyndrome was the sum of NPI score for the individual symptoms, and the total NPI score was the sum of the scores for all symptoms. The presence of NPS was defined as the NPI score of the corresponding subsyndrome being more than 0. Significant NPS was defined as a total NPI score greater than or equal to 4.

### Statistical analysis

Differences in demographics and risk factors were compared between NPS and without NPS subgroups using the chi-square test for categorical variables, one-way analysis of variance for continuous variables. Kendall’s tau-b correlation coefficient was used for the correlation analysis of housing and SES indicators. Then, we examined the association of housing with composite and domain-specific cognitive Z-scores using linear regression, as well as NPI scores (adding a constant of one to obtain a strictly positive distribution) using generalized linear models (GLM) based on gamma distribution and log link function [41]. The optimal model was selected based on the Akaike information criterion (AIC) and Bayesian information criterion (BIC). Binary logistic regression models were used to analyse the association of housing status with the presence of NPS and significant NPS. To investigate the differences in effects of housing status on significant NPS among diverse age groups, sex, ethnicity, SES, and cognitive status in further detail, subgroup analyses were repeated and stratified by age (< 70 versus ≥ 70 years), sex, ethnicity, SES, and cognitive status and used binary logistic model controlling for age, sex, ethnicity, smoking, and drinking status, past medical history of diabetes mellitus, hypertension and hyperlipidaemia, cardiovascular disease, and cognitive status. Then, five models with progressively increased adjustment of all covariates were used for GLM. Model 1 was adjusted for demographics, including age, sex, and ethnicity. Model 2 was further controlled for risk factors including smoking and drinking status, past medical history of diabetes mellitus, hypertension, hyperlipidaemia, and cardiovascular disease. Model 3 was additionally controlling for SES indicators. Model 4 and 5 were further controlled for cognitive status and composite Z-scores, respectively. A sensitivity analysis after excluding the unemployed was conducted. *P*-values less than 0.05 were considered significant for analysis, while *P*-values of less than 0.007 (0.05/7) and less than 0.0125 (0.05/4) were considered significant for cognitive domains and NPS subsyndromes, respectively, after Bonferroni correction. All statistical analyses were performed on R version 4.2.2.

## Results

A total of 911 dementia-free elderly were recruited. Among these, 72 elderly without data on housing and NPS were excluded, leaving 839 elderly in the analysis. Excluded participants were less likely to be female and had a higher proportion of alcohol consumption (*Ps* < 0.05, Table S1). A total of 54 (6.4%) participants were administered in Chinese dialects, including Cantonese, Hokkien and other dialects. A total of 13 (1.5%) participants were unemployed and 325 (38.7%) participants were retired. Demographics of the included 839 dementia-free elderly are shown in Table 1. NPS was present in 165 (19.7%) participants.

**Table 1:** Sample characteristics

Clinical features	Total (*N* = 839)	NPS		*P*
		Yes (*N* = 165)	No (*N* = 674)	
Age, years, mean (SD)^*^	69.8 (6.4)	69.8 (6.2)	69.8 (6.5)	0.94
Female, N (%)^†^	435 (51.8)	86 (52.1)	349 (51.8)	0.94
Ethnicity†				0.51
Chinese, N (%)	292 (34.8)	62 (37.6)	230 (34.1)	
Malay, N (%)	291 (34.7)	51 (30.9)	240 (35.6)	
Indian, N (%)	256 (30.5)	52 (31.5)	204 (30.3)	
Smoking, N (%)^†^	238 (28.4)	47 (28.5)	191 (28.3)	0.97
Drinking, N (%)^†^	47 (5.6)	8 (4.8)	39 (5.8)	0.64
Diabetes mellitus, N (%)^†^	298 (35.5)	63 (38.2)	235 (34.9)	0.43
Hypertension, N (%)^†^	675 (80.5)	119 (72.1)	556 (82.5)	< 0.01
Hyperlipidaemia, N (%)^†^	632 (75.3)	125 (75.8)	507 (75.2)	0.89
Cardiovascular disease, N (%)^†^	81 (9.7)	16 (9.7)	65 (9.6)	0.98
MoCA, mean (SD)^*^	19.3 (5.1)	18.6 (5.5)	19.5 (4.9)	0.04
Composite Z-scores, mean (SD)^*^	0.0 (1.0)	-0.2 (1.1)	0.0 (1.0)	0.02
Cognitive Status^†^				0.32
NCI	265 (31.6)	49 (29.7)	216 (32.0)	
CIND-mild	285 (34.0)	51 (30.9)	234 (34.7)	
CIND-moderate	289 (34.4)	65 (39.4)	224 (33.2)	

*one-way analysis of variance; †chi-square test

*Abbreviations* NPS, Neuropsychiatric symptoms; MoCA, the Montreal Cognitive Assessment; NCI, No cognitive impairment; CIND, Cognitive impairment, no dementia; SD: Standard deviation

Table 2 shows the characteristic of SES indicators according to housing status. Kendall’s tau-b correlation coefficients between housing and individual SES indicators indicated that housing status was correlated with SES indicators (*P*s < 0.01, Table S2).

**Table 2:** Characteristics of SES indicators according to housing status

SES indicator	Housing status			Overall
	1–2 room HDB apartment	3–4 room HDB apartment	Privileged housing	
Education				
No formal education	17 (29.3%)	112 (20.3%)	33 (14.4%)	162 (19.3%)
Primary education	31 (53.4%)	265 (48.0%)	63 (27.5%)	359 (42.8%)
Secondary and above education	10 (17.2%)	175 (31.7%)	133 (58.1%)	318 (37.9%)
Income				
< 1000 S$	53 (91.4%)	396 (71.7%)	138 (60.3%)	587 (70.0%)
1000 ≤ income < 2000 S$	5 (8.6%)	97 (17.6%)	36 (15.7%)	138 (16.4%)
≥ 2000 S$	0 (0%)	53 (9.6%)	51 (22.3%)	104 (12.4%)
Occupation				
Unskilled and unemployed	25 (43.1%)	191 (34.6%)	61 (26.6%)	277 (33.0%)
Semiskilled	12 (20.7%)	108 (19.6%)	44 (19.2%)	164 (19.5%)
Skilled	0 (0%)	27 (4.9%)	24 (10.5%)	51 (6.1%)
Retired	21 (36.2%)	212 (38.4%)	92 (40.2%)	325 (38.7%)

*Abbreviations* HDB, Housing Development Board. Missing data: Income = 10 (1.2%), occupation = 22 (2.6%)

The results showed that higher levels of housing, education, occupation, and income were all significantly associated with better cognitive functioning (*Ps* < 0.001, Table S3).

Compared to living in 1–2 room HDB apartments, living in privileged housing was associated with a lower presence of NPS (OR = 0.49, 95%CI = 0.24 to 0.98, *P* < 0.05) and significant NPS (OR = 0.20, 95%CI = 0.08 to 0.46, *P* < 0.01). No significant association was found between occupation, income, education, and NPS (Table S4).

**Fig. 1:**
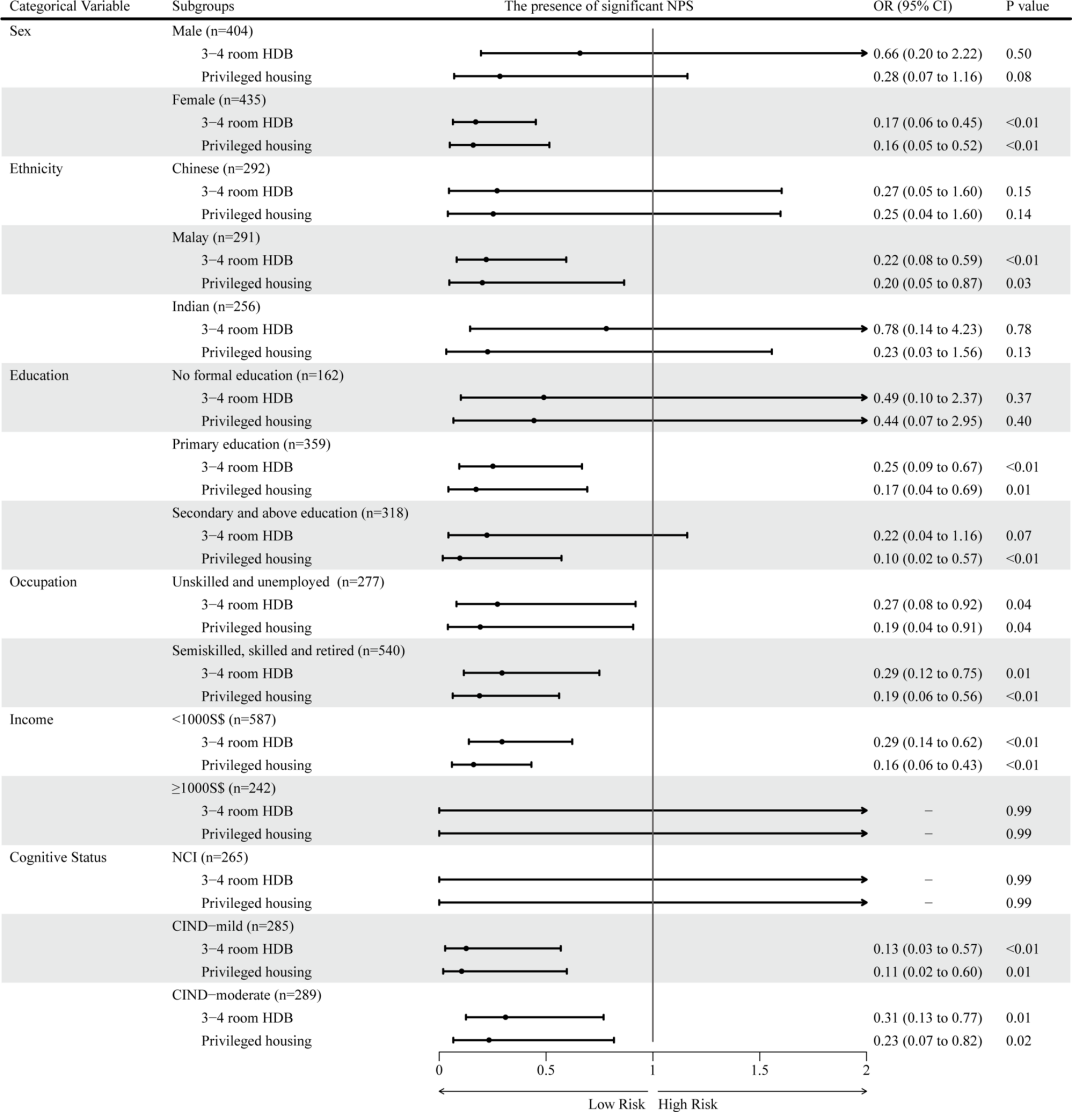
Subgroup analyses for association of housing status with the presence of significant NPS. Reference group was 1–2 room HDB apartment. Models were adjusted for age, sex, ethnicity, smoking and drinking status, past medical history of diabetes mellitus, hypertension, hyperlipidaemia, cardiovascular disease, and cognitive status. Abbreviations: HDB, Housing Development Board; NPS, Neuropsychiatric symptoms; NCI, No cognitive impairment; CIND, Cognitive impairment, no dementia; OR, Odds ratio; CI, Confidence interval

Subgroup analysis indicated a significant association between housing type and the presence of significant NPS in females, those of Malay ethnicity, the more educated, those with lower income, and those diagnosed with CIND (*Ps* < 0.05, Fig. 1), indicating the effect of housing on significant NPS varied by sex, ethnicity, education, income, and cognitive status.

Table S5 shows sample characteristics according to housing type. Adjusting for other SES indicators, the association between housing and total NPI scores remained significant (Table 3). When composite Z-scores were included in the model, housing was still independently associated with the NPI score (privileged housing: *β*=-0.50, 95%CI=-0.95 to -0.04, *P* = 0.032). Participants in privileged housing had lower psychosis subsyndromes (*β*=-0.36, 95%CI=-0.66 to -0.05, *P* = 0.025). The results remained robust after excluding unemployed participants (Table S6).

**Table 3:** Association of housing with NPS and subsyndrome severity

	NPS		Apathy		Affective		Psychosis		Hyperactivity	
	3–4 room HDB apartment* β (95%CI)	Privileged housing * β (95%CI)	3–4 room HDB apartment* β (95%CI)	Privileged housing * β (95%CI)	3–4 room HDB apartment* β (95%CI)	Privileged housing* β (95%CI)	3–4 room HDB apartment* β (95%CI)	Privileged housing* β (95%CI)	3–4 room HDB apartment* β (95%CI)	Privileged housing* β (95%CI)
Univariate+	-0.37 (-0.87, 0.13) *P* = 0.14	**-0.62 (-1.15**,** -0.09)** ***P*** **= 0.023**	-0.14 (-0.46, 0.18) *P* = 0.40	-0.33 (-0.68, 0.01) *P* = 0.06	-0.08 (-0.31, 0.15) *P* = 0.50	-0.12 (-0.36, 0.13) *P* = 0.34	-0.16 (-0.48, 0.16) *P* = 0.33	-0.32 (-0.67, 0.02) *P* = 0.06	-0.30 (-0.63, 0.03) *P* = 0.07	-0.29 (-0.64, 0.05) *P* = 0.10
Model 1	-0.42 (-0.92, 0.08) *P* = 0.10	**-0.67 (-1.21**,** -0.13)** ***P*** **= 0.015**	-0.16 (-0.48, 0.15) *P* = 0.31	-0.34 (-0.67, 0.00) *P* = 0.05	-0.08 (-0.30, 0.15) *P* = 0.51	-0.11 (-0.36, 0.13) *P* = 0.37	-0.26 (-0.57, 0.05) *P* = 0.10	**-0.45 (-0.78**,** -0.11)** ***P*** **= 0.009†**	-0.31 (-0.63, 0.02) *P* = 0.06	-0.31 (-0.66, 0.04) *P* = 0.09
Model 2	-0.39 (-0.88, 0.09) *P* = 0.11	**-0.62 (-1.14**,** -0.09)** ***P*** **= 0.021**	-0.08 (-0.35, 0.20) *P* = 0.59	-0.22 (-0.52, 0.07) *P* = 0.14	-0.07 (-0.30, 0.15) *P* = 0.53	-0.11 (-0.35, 0.14) *P* = 0.39	-0.25 (-0.55, 0.05) *P* = 0.11	**-0.45 (-0.77**,** -0.12)** ***P*** **= 0.007**†	-0.30 (-0.61, 0.01) *P* = 0.06	-0.30 (-0.63, 0.04) *P* = 0.08
Model 3	-0.38 (-0.80, 0.05) *P* = 0.08	**-0.58 (-1.05**,** -0.12)** ***P*** **= 0.015**	-0.05 (-0.31, 0.21) *P* = 0.70	-0.18 (-0.47, 0.10) *P* = 0.21	-0.06 (-0.28, 0.16) *P* = 0.59	-0.09 (-0.33, 0.14) *P* = 0.44	-0.21 (-0.50, 0.07) *P* = 0.14	**-0.40 (-0.71**,** -0.09)** ***P*** **= 0.012†**	**-0.30 (-0.60**,** -0.01)** ***P*** **= 0.04**	-0.31 (-0.64, 0.01) *P* = 0.06
Model 4	-0.29 (-0.70, 0.12) *P* = 0.17	**-0.49 (-0.94**,** -0.04)** ***P*** **= 0.035**	-0.04 (-0.30, 0.22) *P* = 0.77	-0.16 (-0.45, 0.12) *P* = 0.26	-0.04 (-0.25, 0.18) *P* = 0.73	-0.07 (-0.30, 0.17) *P* = 0.57	-0.20 (-0.48, 0.08) *P* = 0.16	**-0.39 (-0.70**,** -0.07)** ***P*** **= 0.015**	-0.22 (-0.51, 0.07) *P* = 0.14	-0.23 (-0.55, 0.09) *P* = 0.16
Model 5	-0.30 (-0.72, 0.11) *P* = 0.15	**-0.50 (-0.95**,** -0.04)** ***P*** **= 0.032**	-0.05 (-0.30, 0.20) *P* = 0.71	-0.17 (-0.45, 0.11) *P* = 0.23	-0.05 (-0.26, 0.16) *P* = 0.64	-0.08 (-0.32, 0.15) *P* = 0.49	-0.17 (-0.46, 0.11) *P* = 0.22	**-0.36 (-0.66**,** -0.05)** ***P*** **= 0.025**	-0.24 (-0.53, 0.05) *P* = 0.10	-0.24 (-0.56, 0.07) *P* = 0.13

*Reference: 1–2 room HDB apartment. †Statistically significant after Bonferroni correction (*P* < 0.0125). Abbreviations: HDB, Housing development board; NPS, Neuropsychiatric symptoms; CI, Confidence interval

Model 1: includes age, sex, and ethnicity as confounders

Model 2: includes Model 1 and risk factors (smoking and drinking status, past medical history of diabetes mellitus, hypertension and hyperlipidaemia, cardiovascular disease) as confounders

Model 3: includes Model 2 and the other three SES indicators as confounders

Model 4: includes Model 3 and cognitive status as confounders

Model 5: includes Model 3, and composite Z-scores as confounders

## Discussion

In this study of a community-dwelling dementia-free elderly population, there was a significant protective effect of better housing on NPS, especially psychosis. Furthermore, housing types were significantly associated with NPS in females, those of Malay ethnicity, the more educated, those with lower income, and those diagnosed with CIND status.

Those living in privileged housing had a lower risk of NPS than those living in the least privileged housing. The results were similar to previous studies in which prior exposure to housing disadvantage was consistently associated with worse mental health [14, 17]. Both environmental characteristics (such as space or temperature) and socioeconomic disadvantages (such as rental costs or health services) of housing could affect mental health and they were interrelated but different [42, 43]. The results showed that housing was correlated with all SES indicators, while the association between housing and overall NPS, with a particular focus on worse psychosis subsyndromes, such as night-time behaviours, was still significant after controlling for SES indicators in our study. Although housing represents a part of SES, the protective effect of housing on NPS was independent of SES and might have other pathogenic mechanisms. One study found that moving into a smaller apartment can increase mental stress [44]. Another study found private renters in unaffordable housing experienced poorer mental health than home purchasers even after adjusting for household income [45]. Housing affordability stress or poor indoor environments may be a direct stressor. Better quality housing not only could abolish housing stressors and improve psychological well-being, but also may reduce mental strain indirectly by increasing resources to endure or address other stressors [44, 46]. Also, housing characteristics such as indoor environments may directly influence sleep comfort and quality, especially among under-resourced populations [47, 48], and lead to night-time behavioural disorders.

We further found that housing types were significantly associated with NPS in females, those of Malay ethnicity, the more educated, those with lower income, and those diagnosed with CIND status. A study found a stronger association between education and cognition among cognitively impaired participants with high educational levels and suggested that those participants may have a more severe Alzheimer’s disease pathology [49]. Previous studies showed that female, Malay ethnicity, household indebtedness, and poor cognitive performance were associated with poor mental health among Asian older adults [23, 50]. Taken together, these findings suggest that housing was an important potentially remediable health determinant, especially among the elderly with a high risk of poor mental health.

Limitations of this study include, firstly that participants who were excluded from the present analyses, were less likely to be female and had a higher proportion of alcohol consumption compared to the included participants. However, despite this non-participation, we still found significant associations of housing with NPS. Second, other housing-related environmental and psychosocial factors, such as home ownership, loneliness, and social support warrant further exploration, although in the present study, no significant interaction between living alone, number of individuals living in the house with housing status on the effect of NPS was found (*P* > 0.05, Table S7). Third, although participants with major psychiatric illnesses or substance abuse disorders were excluded from the study, there may be a small portion of participants on psychotropic medications and therapy. Hence their influence on the association between housing and NPS warrants further investigation. Finally, data collection procedures may introduce information bias that deviates from protocols over the years.

Strengths of this study included the choice of universal and objective housing indicator for reliable and representative results; the use of a multi-ethnic population-based study, extensive neuropsychological and neuropsychiatric tests to determine performance in cognitive and NPS domains; and robustness of the analysis considering the confounding effects.

## Conclusions

Better housing, as an important social determinant of health, may exert significant effects on mental health mainly as an independent or non-SES stressor among dementia-free elderly. Improving housing could be a targeted and effective approach for neuropsychiatric disturbance prevention among the elderly, especially for those at high risk for NPS.

### Electronic supplementary material

Below is the link to the electronic supplementary material.

Supplementary Material 1

## Data Availability

All data relevant to the study are included in the article or uploaded as supplementary information.

## Abbreviations

AIC: Akaike information criterion
AMT: Abbreviated Mental Test
BIC: Bayesian information criterion
CIND: Cognitive impairment, no dementia
DSM-IV: Diagnostic and Statistical Manual of Mental Disorders, Fourth Edition
EDIS: Epidemiology of Dementia in Singapore
GLM: Generalized linear models
HDB: Housing Development Board
MCI: Mild cognitive impairment
MoCA: Montreal Cognitive Assessment
NCI: No cognitive impairment
NPI: Neuropsychiatric inventory
NPS: Neuropsychiatric symptoms
OR: Odds ratio
SD: Standard deviation
SGD: Singaporean dollar
SEED: Singapore Epidemiology of Eye Disease
SES: Socioeconomic status
WAIS-R: Weschler Adult Intelligence Scale-Revised
WMS-R: Weschler Memory Scale-Revised
