# Supplementary material for: Housing status is protective of neuropsychiatric symptoms among dementia-free multi-ethnic Asian elderly
Source: BMC Geriatr. 2024 Aug 23;24:698. doi: 10.1186/s12877-024-05203-x (PMC11342485; doi:10.1186/s12877-024-05203-x)
Supplement: Supplementary file 1 — Supplementary Material 1 [file 12877_2024_5203_MOESM1_ESM.docx]

**Table S1:** Baseline characteristics of included and excluded participants.

| **Clinical Features** | **Included (N=839)** | **Excluded (N=72)** | ***P*** |
| --- | --- | --- | --- |
| Age, years, mean (SD)^*^ | 69.8 (6.4) | 69.8 (5.9) | 0.97 |
| Female, N (%)^†^ | 435 (51.8%) | 24 (33.3%) | <0.01 |
| Ethnicity^†^ |  |  | <0.01 |
| Chinese, N (%) | 292 (34.8%) | 1 (1.4%) |  |
| Malay, N (%) | 291 (34.7%) | 5 (6.9%) |  |
| Indian, N (%) | 256 (30.5%) | 66 (91.7%) |  |
| Smoking, N (%)^†^ | 238 (28.4%) | 19 (26.4%) | 0.72 |
| Drinking, N (%)^†^ | 47 (5.6%) | 9 (12.5%) | 0.03 |
| Diabetes mellitus, N (%)^†^ | 298 (35.5%) | 37 (51.4%) | <0.01 |
| Hypertension, N (%)^†^ | 675 (80.5%) | 56 (77.8%) | 0.58 |
| Hyperlipidaemia, N (%)^†^ | 632 (75.3%) | 60 (83.3%) | 0.13 |
| Cardiovascular disease, N (%)^†^ | 81 (9.7%) | 13 (18.1%) | 0.02 |
| MoCA, mean (SD)^*^ | 19.3 (5.1) | 19.8 (5.7) | 0.45 |
| Cognitive Status^†^ |  |  | 0.81 |
| NCI | 265 (31.6%) | 23 (31.9%) |  |
| CIND-mild | 285 (34.0%) | 22 (30.6%) |  |
| CIND-moderate | 289 (34.4%) | 27 (37.5%) |  |

*one-way analysis of variance; †chi-square test

Abbreviations: MoCA, the Montreal Cognitive Assessment; NCI, No cognitive impairment; CIND, Cognitive impairment, no dementia; SD: Standard deviation.

**Table S2:** Correlations among education, occupation, income, and housing.

|  | **Education** | **Occupation** | **Income** | **Housing** |
| --- | --- | --- | --- | --- |
| Education | 1 | - | - | - |
| Occupation | 0.295* | 1 | - | - |
| Income | 0.299* | 0.419* | 1 | - |
| Housing | 0.218* | 0.095* | 0.168* | 1 |

**P*<0.01. Occupational levels: unskilled and unemployed, retired, semi-skilled, skilled.

**Table S3:** Association of individual SES indicators with cognitive performance.

|  | **Composite Z-scores** | | **Executive function** | | **Attention** | | **Language** | | **Verbal memory** | | **Visual memory** | | **Visuomotor speed** | | **Visuoconstruction** | |
| --- | --- | --- | --- | --- | --- | --- | --- | --- | --- | --- | --- | --- | --- | --- | --- | --- |
|  | ***β***  **(95%CI)** | ***P*** | ***β***  **(95%CI)** | ***P*** | ***β***  **(95%CI)** | ***P*** | ***β***  **(95%CI)** | ***P*** | ***β***  **(95%CI)** | ***P*** | ***β***  **(95%CI)** | ***P*** | ***β***  **(95%CI)** | ***P*** | ***β***  **(95%CI)** | ***P*** |
| **Education** |  |  |  |  |  |  |  |  |  |  |  |  |  |  |  |  |
| No formal education | ref | - | ref | - | ref | - | ref | - | ref | - | ref | - | ref | - | ref | - |
| Primary education | **0.61 (0.48, 0.75)** | **<0.001** | **0.42**  **(0.28, 0.57)** | **<0.001†** | **0.48**  **(0.35, 0.61)** | **<0.001†** | **0.34**  **(0.19, 0.49)** | **<0.001†** | **0.35**  **(0.20, 0.49)** | **<0.001†** | **0.31**  **(0.17, 0.45)** | **<0.001†** | **0.50**  **(0.38, 0.63)** | **<0.001†** | **0.56**  **(0.43, 0.70)** | **<0.001†** |
| Secondary and above education | **1.32 (1.16, 1.47)** | **<0.001** | **0.90**  **(0.74, 1.07)** | **<0.001†** | **1.02**  **(0.87, 1.17)** | **<0.001†** | **0.82**  **(0.65, 1.00)** | **<0.001†** | **1.07**  **(0.90, 1.24)** | **<0.001†** | **0.91**  **(0.75, 1.06)** | **<0.001†** | **1.20**  **(1.06, 1.34)** | **<0.001†** | **1.21**  **(1.06, 1.36)** | **<0.001†** |
| **Occupation** |  |  |  |  |  |  |  |  |  |  |  |  |  |  |  |  |
| Unskilled and unemployed | ref | - | ref | - | ref | - | ref | - | ref | - | ref | - | ref | - | ref | - |
| Semiskilled | **0.19 (0.01, 0.37)** | **0.044** | 0.02 (-0.16, 0.21) | 0.799 | 0.12 (-0.04, 0.29) | 0.148 | 0.16 (-0.02, 0.34) | 0.081 | 0.18 (-0.01, 0.37) | 0.061 | 0.16 (-0.02, 0.33) | 0.074 | **0.27 (0.1, 0.44)** | **0.002** | 0.17 (0.00, 0.35) | **0.056** |
| Skilled | **0.55 (0.28, 0.83)** | **<0.001** | **0.35 (0.08, 0.62)** | **0.010** | **0.43 (0.18, 0.68)** | **<0.001†** | **0.52 (0.25, 0.79)** | **<0.001†** | **0.59 (0.31, 0.87)** | **<0.001†** | **0.42 (0.16, 0.68)** | **0.002†** | **0.58 (0.33, 0.83)** | **<0.001†** | **0.55 (0.28, 0.81)** | **<0.001†** |
| Retired | **0.27 (0.11, 0.43)** | **0.001** | 0.14 (-0.03, 0.3) | 0.100 | **0.18 (0.03, 0.33)** | **0.017** | **0.29 (0.13, 0.45)** | **<0.001†** | **0.18 (0.01, 0.35)** | **0.038** | **0.20 (0.05, 0.36)** | **0.010** | **0.27 (0.12, 0.42)** | **<0.001†** | **0.28 (0.13, 0.44)** | **<0.001†** |
| **Income** |  |  |  |  |  |  |  |  |  |  |  |  |  |  |  |  |
| <1000S$ | ref | - | ref | - | ref | - | ref | - | ref | - | ref | - | ref | - | ref | - |
| 1000 ≤ income < 2000S$ | **0.22 (0.06, 0.37)** | **0.006** | **0.22**  **(0.07, 0.38)** | **0.004†** | **0.20**  **(0.06, 0.34)** | **0.006†** | **0.20**  **(0.04, 0.35)** | **0.013** | 0.14  (-0.02,0.30) | 0.09 | **0.17**  **(0.02, 0.32)** | **0.023** | **0.18**  **(0.04, 0.32)** | **0.013** | 0.09  (-0.06,0.25) | 0.22 |
| ≥2000S$ | **0.40 (0.23, 0.58)** | **<0.001** | **0.30**  **(0.13, 0.48)** | **<0.001†** | **0.31**  **(0.14, 0.47)** | **<0.001****†** | **0.33**  **(0.15, 0.50)** | **<0.001†** | **0.39**  **(0.21, 0.57)** | **<0.001†** | **0.34**  **(0.17, 0.51)** | **<0.001†** | **0.40**  **(0.24,0.57)** | **<0.001†** | **0.31**  **(0.14, 0.49)** | **<0.001†** |
| **Housing** |  |  |  |  |  |  |  |  |  |  |  |  |  |  |  |  |
| 1-2 room HDB | ref | - | ref | - | ref | - | ref | - | ref | - | ref | - | ref | - | ref | - |
| 3-4 room HDB | **0.26 (0.04, 0.48)** | **0.021** | 0.14  (-0.07, 0.36) | 0.20 | **0.27**  **(0.07, 0.47)** | **0.009** | 0.19  (-0.03,0.41) | 0.09 | 0.16  (-0.07,0.38) | 0.17 | 0.10  (-0.11,0.31) | 0.35 | **0.28**  **(0.08, 0.48)** | **0.006†** | **0.30**  **(0.08, 0.51)** | **0.006†** |
| privileged housing | **0.45 (0.22, 0.69)** | **<0.001** | **0.32**  **(0.09, 0.55)** | **0.007** | **0.42**  **(0.20, 0.63)** | **<0.001†** | **0.37**  **(0.14, 0.61)** | **<0.002†** | **0.42**  **(0.18, 0.66)** | **0.001†** | **0.28**  **(0.06, 0.51)** | **0.014** | **0.53**  **(0.32, 0.75)** | **<0.001†** | **0.43**  **(0.21, 0.66)** | **<0.001†** |

†Statistically significant after Bonferroni correction (*P*<0.007).

Adjustment for age, sex, ethnicity, smoking and drinking status, past medical history of diabetes mellitus, hypertension, hyperlipidemia, and cardiovascular disease.

**Table S4:** Association of socioeconomic indicators with NPS.

| SES indicator | The presence of NPS | | The presence of significant NPS | |
| --- | --- | --- | --- | --- |
|  | OR (95%CI) | *P* | OR (95%CI) | *P* |
| Education |  |  |  |  |
| No formal education | reference | - | reference | - |
| Primary education | 1.34 (0.80, 2.24) | 0.27 | 1.00 (0.51, 1.97) | 0.99 |
| Secondary and above education | 1.08 (0.60, 1.95) | 0.79 | 0.85 (0.40, 1.82) | 0.68 |
| Occupation |  |  |  |  |
| Unskilled and unemployed | reference | - | reference | - |
| Semiskilled | 0.76 (0.42, 1.38) | 0.37 | 0.83 (0.37, 1.86) | 0.65 |
| Skilled | 0.74 (0.31, 1.80) | 0.51 | 0.49 (0.13, 1.92) | 0.31 |
| Retired | 1.22 (0.74, 2.03) | 0.43 | 1.57 (0.80, 3.07) | 0.19 |
| Income |  |  |  |  |
| <1000S$ | reference | - | reference | - |
| 1000 ≤ income < 2000S$ | 0.70 (0.41, 1.19) | 0.18 | 0.56 (0.27, 1.16) | 0.12 |
| ≥2000S$ | 0.69 (0.38, 1.26) | 0.23 | 0.53 (0.23, 1.22) | 0.14 |
| Housing |  |  |  |  |
| 1-2 room HDB | reference | - | reference | - |
| 3-4 room HDB | 0.58 (0.31, 1.09) | 0.09 | **0.31 (0.15, 0.64)** | **<0.01*** |
| Privileged housing | **0.49 (0.24, 0.98)** | **<0.05*** | **0.20 (0.08, 0.46)** | **<0.01*** |

**P*<0.05. Abbreviations: CI, confidence interval; HDB, Housing Development Board; NPS, Neuropsychiatric symptoms; OR, odds ratio. Adjustment for age, gender, ethnicity, the status of smoking and drinking, past medical history of diabetes mellitus, hypertension, hyperlipidaemia, cardiovascular disease, and cognitive status.

**Table S5:** Sample characteristics of the sample according to housing categories (n=839)

| **Clinical Features** | **Housing** | | | ***P*** |
| --- | --- | --- | --- | --- |
|  | **1-2 room HDB (n=58)** | **3-4 room HDB (n=552)** | **Privileged housing**  **(N=229)** |  |
| Age, years, mean (SD)^*^ | 71.7 (6.6) | 69.6 (6.4) | 69.8 (6.3) | 0.06 |
| Female, N (%)^†^ | 34 (58.6) | 296 (53.6) | 105 (45.9) | 0.08 |
| Ethnicity^†^ |  |  |  | <0.01 |
| Chinese, N (%) | 8 (13.8) | 185 (33.5) | 99 (43.2) |  |
| Malay, N (%) | 37 (63.8) | 204 (37.0) | 50 (21.8) |  |
| Indian, N (%) | 13 (22.4) | 163 (29.5) | 80 (34.9) |  |
| Smoking, N (%)^†^ | 17 (29.3) | 158 (28.6) | 63 (27.5) | 0.94 |
| Drinking, N (%)^†^ | 2 (3.4) | 30 (5.4) | 15 (6.6) | 0.63 |
| Diabetes mellitus, N (%)^†^ | 24 (41.4) | 194 (35.1) | 80 (34.9) | 0.63 |
| Hypertension, N (%)^†^ | 49 (84.5) | 451 (81.7) | 175 (76.4) | 0.17 |
| Hyperlipidaemia, N (%)^†^ | 45 (77.6) | 418 (75.7) | 169 (73.8) | 0.78 |
| Cardiovascular, N (%)^†^ | 7 (12.1) | 47 (8.5) | 27 (11.8) | 0.30 |
| MoCA, mean (SD)^*^ | 16.2 (4.1) | 19.1 (5.0) | 20.6 (5.0) | <0.01 |
| Composite Z-score, mean (SD)^*^ | -0.6 (0.9) | 0.0 (1.0) | 0.3 (1.0) | <0.01 |
| Cognitive Status^†^ |  |  |  | <0.01 |
| NCI | 7 (12.1) | 171 (31.0) | 87 (38.0) |  |
| CIND-mild | 12 (20.7) | 195 (35.3) | 78 (34.1) |  |
| CIND-moderate | 39 (67.2) | 186 (33.7) | 64 (27.9) |  |

* one-way analysis of variance; †chi-square test

Abbreviations: HDB, Housing Development Board; MoCA, the Montreal Cognitive Assessment; NCI, No cognitive impairment; CIND, Cognitive impairment, no dementia; SD: Standard deviation.

**Table S6:** Association of occupation with NPS after excluding unemployed participants.

| SES indicator | The presence of NPS | | The presence of significant NPS | |
| --- | --- | --- | --- | --- |
|  | OR (95%CI) | *P* | OR (95%CI) | *P* |
| Occupation |  |  |  |  |
| Unskilled | reference | - | reference | - |
| Semiskilled | 0.73 (0.40, 1.35) | 0.32 | 0.87 (0.38, 1.99) | 0.73 |
| Skilled | 0.72 (0.29, 1.77) | 0.47 | 0.52 (0.13, 2.07) | 0.36 |
| Retired | 1.19 (0.71, 2.01) | 0.50 | 1.67 (0.83, 3.33) | 0.15 |

**P*<0.05. Abbreviations: CI, confidence interval; HDB, Housing Development Board; NPS, Neuropsychiatric symptoms; OR, odds ratio. Adjustment for age, gender, ethnicity, the status of smoking and drinking, past medical history of diabetes mellitus, hypertension, hyperlipidaemia, cardiovascular disease, and cognitive status.

**Table** **S7:** Interaction effect of living alone, number of individuals living in the house and housing on the effect of NPS severity.

|  | ***β* (95%CI)** | **P** |
| --- | --- | --- |
| **Interaction between housing**† **and living alone**†† | | |
| 3-4 room HDB apartment*Living alone | -0.07 (-1.21, 1.07) | 0.91 |
| Privileged housing*Living alone | -0.21 (-1.75, 1.34) | 0.79 |
| **Interaction between housing and number of individuals living in the house** | | |
| 3-4 room HDB apartment*Number of individuals living in the house | -0.02 (-0.32, 0.29) | 0.92 |
| Privileged housing*Number of individuals living in the house | 0.03 (-0.28, 0.34) | 0.86 |

†Reference: 1-2 room HDB apartment. ††Reference: didn’t living alone. Abbreviations: HDB, Housing Development Board; NPS, Neuropsychiatric symptoms; CI, Confidence interval. Adjustment for age, gender, ethnicity, the status of smoking and drinking, past medical history of diabetes mellitus, hypertension, hyperlipidaemia, cardiovascular disease, the other three SES indicators and cognitive status.
